# Predictive value of creatinine-cystatin C ratio for mortality and technique failure in anuric peritoneal dialysis patients

**DOI:** 10.1080/0886022X.2024.2444389

**Published:** 2025-01-02

**Authors:** Zhihong Zhang, Tingting Zhou, Man Zhang, Shuiqin Cheng

**Affiliations:** National Clinical Research Center of Kidney Diseases, Jinling Hospital, Nanjing University School of Medicine, Nanjing, Jiangsu, China

**Keywords:** Peritoneal dialysis, death, technique failure, ratio of creatinine to cystatin C

## Abstract

**Objectives:**

Both serum creatinine and cystatin C serve as dependable markers of renal function and have demonstrated a correlation with clinical outcomes in diverse conditions, particularly through the ratio of creatinine to cystatin C (Cr/CysC). Anuric patients undergoing peritoneal dialysis (PD) depend entirely on the clearance of peritoneal solutes. This research posits that the Cr/CysC ratio may predict all-cause mortality and technique failure in anuric PD patients.

**Methods:**

A cohort of 224 anuric PD patients was recruited from January 1, 2014, to December 31, 2019, with follow-up extending until December 31, 2023. The Cr/CysC ratio was computed by dividing the serum creatinine concentration by the cystatin C concentration obtained on the same day. We evaluated the relationship between the Cr/CysC ratio and patient survival, as well as technique failure, utilizing Cox regression and competing risk analyses.

**Results:**

The average age of the participants was 50.46 ± 12.63 years, with 99 individuals (44.2%) being male. Among all subjects, the Cr/CysC ratio was found to be 1.85 ± 0.56. After controlling for potential confounders, Cox proportional hazards models revealed that the Cr/CysC ratio was significantly linked to the risk of all-cause mortality and technique failure (HR = 0.330, 95% CI 0.124-0.881, *p* = 0.027; HR = 0.440, 95% CI 0.267-0.726, *p* = 0.002). Importantly, the significant associations observed in the Cox regression analysis persisted even after accounting for competing risks.

**Conclusion:**

The Cr/CysC ratio at baseline was recognized as an independent predictor of all-cause mortality and technique failure in anuric PD patients.

## Introduction

Peritoneal dialysis (PD) serves as an essential form of renal replacement therapy for individuals suffering from end-stage kidney disease (ESKD). In 2023, the Chinese National Renal Data System reported that the number of patients undergoing PD was 152,745 and continues to grow. Globally, the prevalence of PD centers increased from 1.4 per million population (pmp) to 1.6 pmp, and PD accounts for 9% of all kidney replacement therapy (KRT) and 11% of dialysis treatments [1, 2]. The ERA Registry Annual Report 2021 indicated that the prevalence of kidney replacement therapy reached 1,040 pmp, with 5% of those patients undergoing PD [3]. Nevertheless, the long-term survival rates for patients receiving PD tend to be comparatively low [4]. During PD, glucose absorption has been associated with the emergence of various metabolic and cardiovascular issues, potentially leading to elevated technique failure and mortality rates noted in some research [5–7].

Creatinine, primarily derived from muscle tissue, has its serum levels affected by the total muscle mass in the body. At present, the Chronic Kidney Disease Epidemiology Collaboration (CKD-EPI) equation is the most frequently utilized method in clinical settings among the numerous creatinine-based equations [8]. In patients undergoing PD, residual renal function (RRF) is characterized by the average clearance rates of urea and creatinine over a 24-h period [9]. Additionally, serum cystatin C – a 13-kD cysteine proteinase inhibitor found in all nucleated cells – remains less influenced by the mass of skeletal muscle. Since serum creatinine concentrations are more reliant on muscle mass compared to cystatin C levels, the serum creatinine-cystatin C (Cr/CysC) ratio has been suggested to relate to muscle mass across different patient populations, which include older adults [10], cancer patients [11], and those with chronic kidney disease (CKD) not undergoing dialysis [12]. Studies have indicated that, assuming the Cr/CysC ratio reflects muscle mass, there exists a correlation between this ratio and patient outcomes across several diseases [13, 14]. The cohort study from the National Health and Nutrition Examination Survey (NHANES), conducted between 1999 and 2002, found that among adult community residents, each 0.01 increase in the Cr/CysC ratio was associated with a 2% reduction in the risk of death (aHR, 0.98; 95% CI, 0.98–0.99, *p* < 0.001)[15]. Additionally, a single-center retrospective cohort study involving 1,141 adult patients with stage 1–5 non-dialysis CKD was conducted between 2016 and 2018, revealing that lower values of the Cr/CysC ratio, assessed both as continuous and categorical variables, independently predicted mortality [16]. Another retrospective cohort study included 1,588 patients with acute kidney injury (AKI) who underwent continuous kidney replacement therapy (CKRT). Multivariable Cox proportional hazards regression analyses indicated that the risk of 30- and 90-day mortality decreased successively across quartiles of increasing Cr/CysC ratio. Furthermore, when the Cr/CysC ratio was evaluated using cubic spline analyses, both 30- and 90-day mortality risks were found to be lower at higher Cr/CysC ratios [17].

However, the connection between the baseline Cr/CysC ratio and all-cause mortality, along with technique failure in patients undergoing PD, has not been extensively explored. To address this, we carried out a retrospective cohort study with anuric PD patients at our institution to investigate the link between the Cr/CysC ratio and negative outcomes.

## Materials and methods

### Study population

This research involved patients who had PD catheterization at our facility between January 1, 2014, and December 31, 2019. All participants were treated with either continuous ambulatory peritoneal dialysis (CAPD) or daytime ambulatory peritoneal dialysis (DAPD). To qualify, patients needed to have been anuric for over three months and have undergone PD for a minimum of three months. Exclusion criteria included: (1) individuals younger than 18 years, (2) those who transitioned from permanent hemodialysis (HD), (3) irregular thyroid function, (4) prolonged use of hormones or cimetidine, (5) the presence of malignant tumors, (6) peritonitis occurring within 4 weeks prior to enrollment, or (7) insufficient adherence, characterized by not attending regular follow-ups. The study adhered to the Declaration of Helsinki principles and was approved by the Ethics Committee of Jinling Hospital. The Ethics Committee granted a waiver for written informed consent due to the study’s retrospective design.

### Study outcomes

The research centered on two main events of significance: mortality from any cause and the failure of the technique, as measured by the Cr/CysC ratio in the participants. The latter was expressly characterized as a confirmed shift to HD for any reason. Every patient was observed until the end of PD, death, the switch to HD or renal transplantation, loss to follow-up, or up to December 31, 2023.

### Data collection

Demographic information about the participants was gathered, which included age, sex, and body mass index (BMI), as well as the primary reason for ESKD. Various laboratory tests were performed to assess levels of hemoglobin, serum albumin, high-sensitivity C-reactive protein (hs-CRP), uric acid, creatinine, cystatin C, calcium, and phosphorus. Following centrifugation, serum samples were analyzed biochemically within one hour of collection. Serum creatinine levels were measured using an autoanalyzer (Siemens Advia 1800, Siemens Healthcare GmbH, Henkestr, Germany), while Cystatin C levels were quantified via immunonephelometry, calibrated against the reference standard. Furthermore, a 24-h sample of dialysate was collected for subsequent analysis. Both urea, creatinine, and glucose concentrations in the 24-h dialysate and serum were evaluated concurrently. The total weekly urea clearance (Kt/V) and normalized protein catabolic rate (nPCR) were determined using established procedures. Following the guidelines of the International Society for Peritoneal Dialysis (ISPD), a standard peritoneal equilibrium test (PET) was executed: dialysate samples were taken at 0, 2, and 4 h, while a blood sample was drawn at 120 min. All samples of blood and dialysate underwent analysis within a 24-h timeframe after collection. The classification of peritoneal solute transport was based on the 4-h dialysate-to-plasma ratio for creatinine (D/Pcr). Specifically, a D/Pcr value of less than 0.50 indicated low transport, while a value ranging from 0.50 to 0.65 signified low-average transport. High-average transport was defined by D/Pcr values between 0.65 and 0.82, and a value exceeding 0.82 was categorized as high transport. The Cr/CysC ratio was derived by dividing the serum creatinine level (mg/dL) by the cystatin C level (mg/L) obtained on the same day. Patients were divided into two groups based on the mean baseline Cr/CysC ratio: those with a low Cr/CysC ratio (Cr/CysC < 1.85) and those with a high Cr/CysC ratio (Cr/CysC ≥ 1.85). The data collected were reviewed from electronic medical records.

### Statistical analysis

The Kolmogorov-Smirnov test was utilized to evaluate the normal distribution of continuous variables. Characteristics of the patients were summarized using means, standard deviations (SD), medians, interquartile ranges, counts, and percentages, based on the type of data. Comparisons between groups were performed using Student’s *t*-test, one-way analysis of variance, Kruskal–Wallis test, or chi-square tests, as deemed suitable. The correlation between the Cr/CysC ratio and other variables was analyzed using Pearson correlation analysis. Patient survival and technique failure were examined through both conventional Cox regression and the competing risk model established by Fine and Gray. Those patients who remained alive at the conclusion of the study were treated as censored consistently. For a comprehensive understanding of our classification of censoring and competing risks within both the Cox and Fine and Gray frameworks, as well as the incidents of mortality and technique failure, kindly refer to Table 1. We applied restricted cubic spline (RCS) models to assess the linear association between the Cr/CysC ratio and all-cause mortality or technique failure. We then evaluated all possible confounders in univariate analyses (with and without accounting for competing risks), choosing those with a *p*-value below 0.10 for inclusion in the multivariate model. To compare distributions of patient survival and technique failure, we utilized the Kaplan–Meier survival technique and the Log-rank test. The receiver operating characteristic (ROC) analysis was performed to determine the area under the ROC curve (AUC) for the Cr/CysC ratio related to the occurrence of death from any cause or technique failure. We concentrated on the comparison of the incidence rates of all-cause mortality and technique failure between two groups *via* the chi-square test. The parameters considered included an effect size of 0.3, an alpha error probability of 0.05, one degree of freedom, and a sample size of 208, leading to a power of 0.991. Analyses were performed utilizing STATA version 14 (STATA Corp., College Station, TX, USA) along with R language (version 4.0.2; R Foundation for Statistical Computing, Vienna, Austria). Two-sided P-values were computed, and a statistical significance threshold was set at *p* < 0.05.

**Table 1.** Definition used for censoring, events and competing risks.

	Event	Competing risk	Censored
Cox regression analysis	D	–	TF + T + L + A
	TF	–	D + T + L + A
Competing risk analysis	D	TF + T + L	A
	TF	D + T + L	A

D: death for any cause; TF: technique failure; T: transplantation; L: loss of follow-up; A: alive at the end of the study.

## Results

### Patient Characteristics

**Figure 1.**
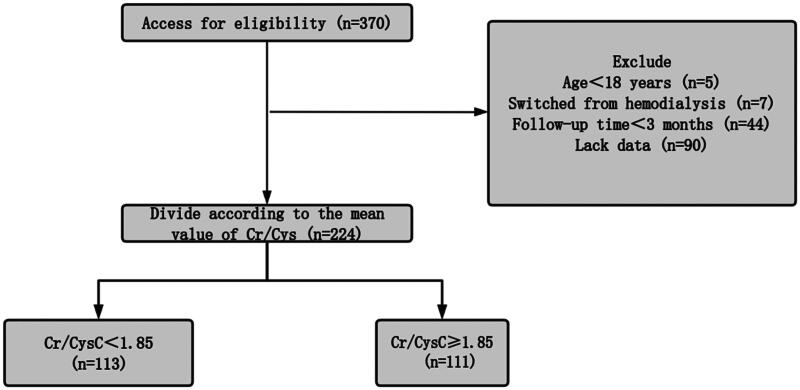
Flow chart of the participants in the study cohort.

Out of 370 PD patients considered for this research, 146 were not eligible, leading to the inclusion of 224 patients in the ultimate analysis (see Figure 1). The cohort’s baseline characteristics are outlined in Table 2. The average age of the participants was 50.46 ± 12.63 years, with 99 individuals (44.2%) being male. The predominant primary cause of ESKD identified was glomerulonephritis (82.1%). For all participants included in the study, the Cr/CysC ratio was found to be 1.85 ± 0.56. Those in the high Cr/CysC ratio group demonstrated elevated levels of serum creatinine, uric acid, and phosphorus, alongside lower levels of cystatin C and total Kt/V. The median follow-up period for these patients was 29 months (ranging from 15 to 53 months). Among the patients, 74 remained on PD, 83 transitioned to HD, 38 received kidney transplants, 3 were lost to follow-up, and 26 patients passed away.

**Table 2.** Baseline characteristics of 224 PD patients.

Characteristics	Total (*n* = 224)	Cr/CysC < 1.85 (*n* = 113)	Cr/CysC ≥ 1.85 (*n* = 111)	*P*-value
Demographics				
Age (year)	50.46 ± 12.63	51.79 ± 13.47	48.88 ± 11.42	0.086
Men *n* (%)	99 (44.2)	47 (41.6)	52 (46.8)	0.569
history of CVD *n* (%)	26 (11.6)	15 (13.3)	11 (9.9)	0.533
BMI (kg/m^2^)	20.62 ± 2.58	20.31 ± 2.60	20.97 ± 2.51	0.063
Primary kidney disease				0.833
Chronic glomerulonephritis *n* (%)	184 (82.1)	88 (77.8)	96 (86.4)	
Hypertensive nephropathy *n* (%)	7 (3.2)	3 (2.8)	4 (3.6)	
Diabetic nephropathy *n* (%)	11 (4.9)	8 (7.0)	3(2.8)	
Other *n* (%)	22 (9.8)	14 (12.4)	8(7.2)	
Laboratory variables				
Hemoglobin (g/L)	101.75 ± 19.75	103.14 ± 18.94	100.35 ± 20.52	0.299
hs-CRP (mg/L)	2.35 (0.30, 5.10)	1.80(0.10, 4.95)	2.85(0.70, 5.55)	0.075
Serum creatinine (mg/dL)	12.41 ± 3.44	10.84 ± 2.85	14.01 ± 3.26	<0.001
Serum cystatin C (mg/L)	6.71 ± 1.90	7.48 ± 2.15	5.96 ± 1.23	<0.001
Serum urea nitrogen (mg/dL)	62.99 ± 20.87	60.35 ± 21.16	65.67 ± 20.33	0.056
Serum uric acid (μmol/L)	436.74 ± 94.31	425.29 ± 95.53	448.30 ± 92.05	0.033
Corrected calcium (mmol/L)	2.29 ± 0.41	2.31 ± 0.48	2.28 ± 0.32	0.397
Serum phosphorus (mmol/L)	2.00 ± 0.67	1.80 ± 0.52	2.20 ± 0.74	<0.001
Serum albumin (g/L)	38.91 ± 4.63	38.63 ± 4.89	39.19 ± 4.35	0.288
Total cholesterol (mmol/L)	4.75 ± 1.15	4.75 ± 1.16	4.74 ± 1.14	0.767
Triglycerides (mmol/L)	1.63 (1.16, 2.40)	1.61 (1.12, 2.33)	1.67 (1.25, 2.50)	0.435
Fasting blood glucose (mmol/L)	5.52 ± 1.29	5.62 ± 1.59	5.41 ± 0.89	0.413
Total Kt/V	1.70 ± 0.41	1.76 ± 0.47	1.65 ± 0.33	0.029
nPCR (g/kg/d)	0.92 ± 0.19	0.93 ± 0.21	0.91 ± 0.17	0.466
H/HA n (%)	83 (37.1)	47 (41.5)	36 (32.4)	0.189
Cr/CysC	1.85 ± 0.56	1.45 ± 0.29	2.35 ± 0.37	<0.001
Outcomes				
Death for any cause	26 (11.6)	19 (16.8)	7 (6.3)	0.025
Technique failure	83 (37.1)	51 (45.1)	32 (28.8)	0.028

Cr/CysC: serum creatinine-cystatin C ratio; BMI: body mass index; hs-CRP: high-sensitivity C-reactive protein; Total Kt/V: weekly total urea clearance; nPCR: normalized protein catabolic rate; H: peritoneal transport type high; HA: peritoneal transport type high average.

### Correlation between Cr/CysC ratio and other parameters

Table 3 illustrates the relationships between the Cr/CysC ratio and various other parameters. A positive correlation was observed between the Cr/CysC ratio and BMI, serum uric acid, and phosphorus. Conversely, age and total Kt/V showed a negative correlation with the Cr/CysC ratio.

**Table 3.** Pearson correlation analysis of factors associated with Cr/CysC.

Variables	*r*	*P*-value
Age (years)	−0.018	0.008
BMI (kg/m^2^)	0.169	0.016
Albumin (g/L)	0.103	0.130
Serum uric acid (μmol/L)	0.188	0.006
Serum phosphorus (mmol/L)	0.311	<0.001
hs-CRP (mg/L)	0.428	0.523
Total Kt/V	−0.206	0.002
nPCR	−0.033	0.632

Cr/CysC: serum creatinine-cystatin C ratio; BMI: body mass index; hs-CRP: high-sensitivity C-reactive protein; Total Kt/V: weekly total urea clearance.

### Association between Cr/CysC ratio and all-cause mortality or technique failure

**Figure 2.**
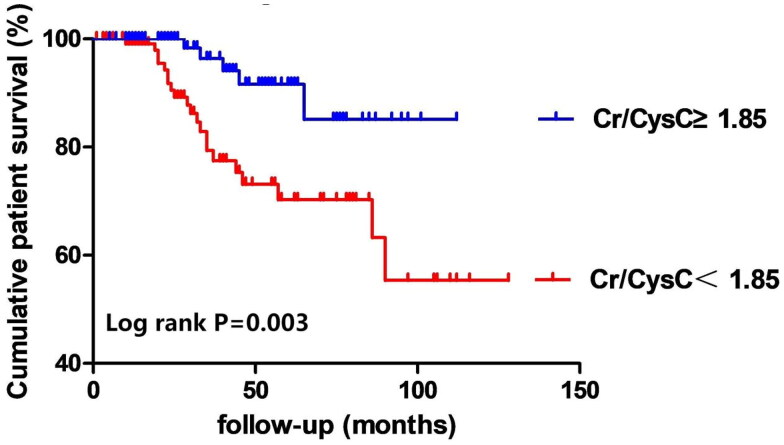
Cumulative all-cause mortality curves. Cr/CysC: serum creatinine-cystatin C ratio.

**Figure 3.**
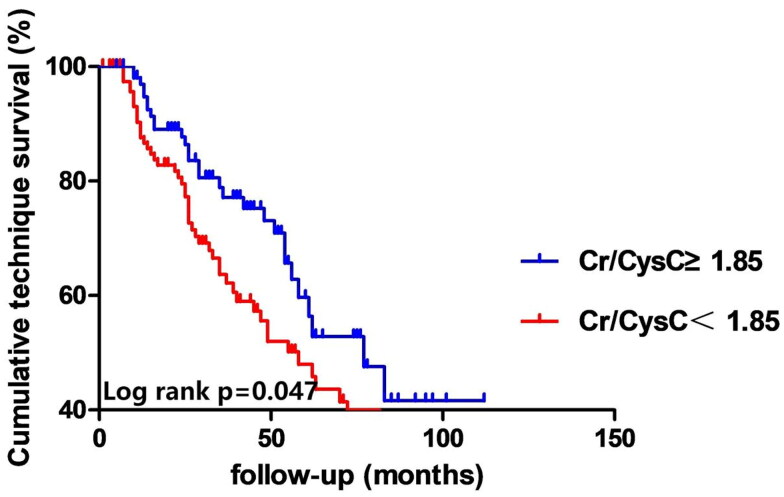
Cumulative technique failure curves. Cr/CysC: serum creatinine-cystatin C ratio.

**Figure 4.**
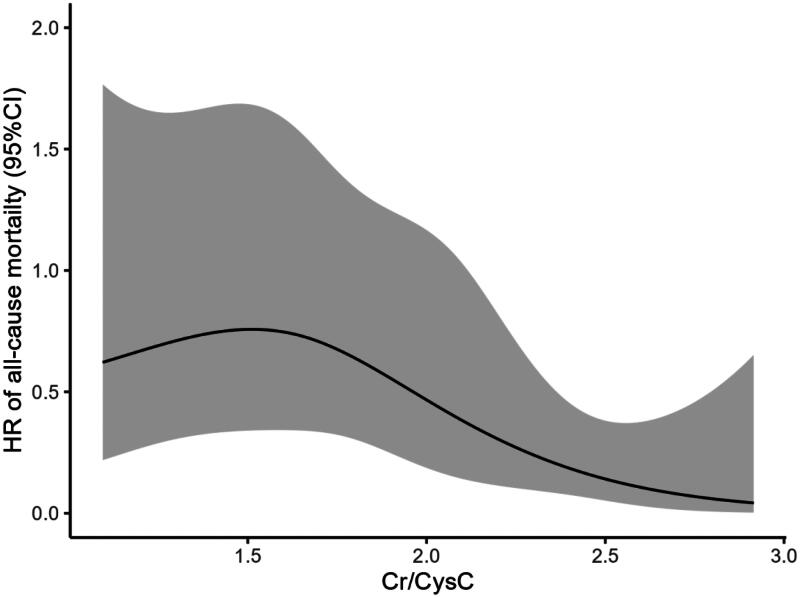
Cubic spline model showing the relationship between Cr/CysC ratio and all-cause mortality.

**Figure 5.**
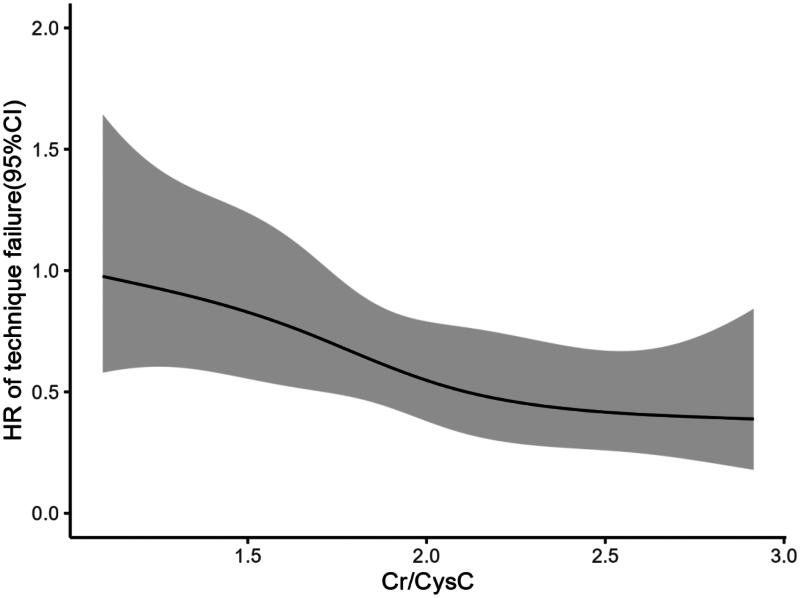
Cubic spline model showing the relationship between Cr/CysC ratio and technique failure.

In the group with a low Cr/CysC ratio, the rates of all-cause mortality and technique failure were observed to be 16.8% and 45.1%, respectively. Conversely, in the group with a high Cr/CysC ratio, these figures dropped to 6.3% and 28.8% (see Table 2). Analysis using the Kaplan-Meier method demonstrated that the rates of all-cause mortality and technique failure were notably reduced in the high Cr/CysC ratio group in comparison to the low Cr/CysC ratio group (refer to Figures 2 and 3). The Cr/CysC ratio exhibited a correlation with patient survival in both analytical models, even after accounting for potential confounding variables: Cox regression analysis (HR 0.330; 95% CI 0.124–0.881) and competing risk analysis (HR 0.465; 95% CI 0.228–0.942). When assessing technique failure based on the Cr/CysC ratio, the high Cr/CysC ratio category displayed a diminished risk of technique failure compared to the low Cr/CysC ratio category. Furthermore, the Cr/CysC ratio was linked to technique failure in both models following adjustments for confounding factors: Cox regression analysis (HR 0.440; 95% CI 0.267–0.726) and competing risk analysis (HR 0.554; 95% CI 0.371–0.828). When evaluated as a dichotomous variable, the association remained statistically significant in the adjusted models (see Tables 4 and 5). Moreover, cubic spline analyses further explored the links between the Cr/CysC ratio and patient outcomes, revealing a progressive decline in risks for both all-cause mortality and technique failure with an increase in Cr/CysC ratio (see Figures 4 and 5).

**Table 4.** Multivariate analysis of Cr/CysC in association with all-cause mortality.

	COX		FINE AND GRAY	
	HR (95% CI)		HR (95% CI)	
	Univariate	Multivariate	Univariate	Multivariate
Continuous variable				
Cr/CysC	0.356 (0.158–0.800)*	0.330 (0.124–0.881)*	0.435 (0.251–0.753)*	0.465 (0.228–0.942)*
Dichotomous variable				
Cr/CysC < 1.85	Reference		Reference	
Cr/CysC ≥ 1.85	0.257 (0.097–0.683)*	0.199 (0.063–0.629)*	0.253 (0.096–0.666)*	0.244(0.076–0.785)*

Cr/CysC: serum creatinine-cystatin C ratio; HR: hazard ratio; 95% CI: 95% confidence interval. Adjusted for age, sex, history of diabetes, albumin, uric acid, total cholesterol, phosphorus, hemoglobin, and Total Kt/V. *p < 0.05 was considered statistically significant.

**Table 5.** Multivariate analysis of Cr/CysC in association with technique failure.

	COX		FINE AND GRAY	
	HR (95% CI)		HR (95% CI)	
	Univariate	Multivariate	Univariate	Multivariate
Continuous variable				
Cr/CysC	0.531 (0.362–0.779)*	0.440(0.267–0.726)*	0.562 (0.392–0.806)*	0.554 (0.371–0.828)*
Dichotomous variable				
Cr/CysC < 1.85	Reference		Reference	
Cr/CysC ≥ 1.85	0.595 (0.400–0.884)*	0.402 (0.221–0.730)*	0.554 (0.354–0.884)*	0.524 (0.316–0.867)*

Cr/CysC: serum creatinine-cystatin C ratio; HR: hazard ratio; 95% CI: 95% confidence interval. Adjusted for age, sex, history of diabetes, albumin, uric acid, total cholesterol, phosphorus, hemoglobin, hs-CRP, and total Kt/V. *p < 0.05 was considered statistically significant.

### ROC curves for Cr/CysC ratio on the presence of death or technique failure

**Figure 6.**
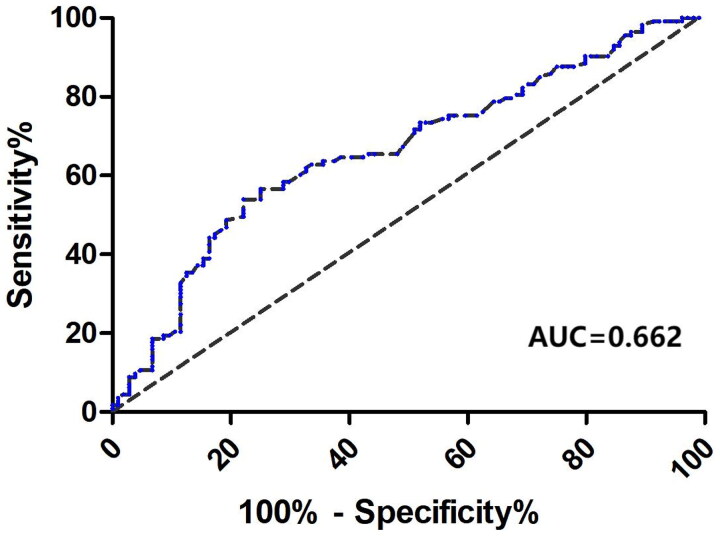
Receiver operating characteristic (ROC) curve and area under the ROC curve (AUC) of Cr/CysC ratio for the presence of death or technique failure.

The endpoint for death from all causes yielded an AUC for the Cr/CysC ratio of 0.652 (95% CI: 0.552, 0.753), with a *p*-value of 0.003. Similarly, technical failure as an endpoint resulted in an AUC for the Cr/CysC ratio of 0.613 (95% CI: 0.536, 0.690), with a *p*-value of 0.004. The AUC for the Cr/CysC ratio in predicting the occurrence of death or technique failure was calculated to be 0.662 (95% CI: 0.590–0.734, *p* < 0.001). The best cutoff value for the Cr/CysC ratio related to the incidence of death or technique failure was identified as 2.05, resulting in a Youden index of 0.319, with a sensitivity of 54.0% and a specificity of 75.0% (Figure 6).

## Discussion

In this research, the Cr/CysC ratio was discovered to be linked with both overall mortality and the failure of procedures in anuric patients undergoing PD. Patients were classified based on the mean value of the Cr/CysC ratio recorded at the beginning of the study, which indicated that a higher ratio was significantly associated with a lower risk of overall mortality and procedural failure. This correlation retained its statistical significance even after accounting for various demographic factors and previously recognized indicators of dialysis adequacy, including total Kt/V. One possible reason for the observed connection between the Cr/CysC ratio and both mortality and technique failure in those with anuric PD might be that this ratio serves as a proxy for muscle mass, which is thought to affect outcomes in different patient groups [16, 17]. Those suffering from CKD are especially vulnerable to the dangers posed by muscle wasting. There is a notable prevalence of muscle wasting in the CKD population, particularly among individuals diagnosed with ESKD [18, 19]. Factors that contribute to this issue during the advancement of CKD include inflammation, acidosis, malnutrition, issues with insulin signaling, lowered levels of sex or growth hormones, excessive angiotensin II, and the buildup of uremic toxins [20, 21]. Patients undergoing dialysis face a heightened risk of having low muscle mass, which can result in frailty, increased falls, fractures, reduced quality of life, as well as a higher probability of infections and early mortality. The Cr/CysC ratio is a readily accessible alternative measure for evaluating muscle mass. Numerous investigations have shown significant associations between the Cr/CysC ratio and muscle mass in elderly patients, those with chronic obstructive pulmonary disease, and cancer patients, along with the prognostic importance of the Cr/CysC ratio in individuals with non-dialysis CKD [10–14]. The results indicate that the relationship involving the Cr/CysC ratio and outcomes could likewise be relevant for patients receiving PD. Furthermore, we found that the Cr/CysC ratio’s relationship to all-cause mortality, illustrated in the restricted cubic spline plot presented in this article, contrasts with results from other investigations. This variation may stem from differences in the populations examined. Certain studies have suggested that in the non-CKD cohort, the mortality risk displays a U-shaped pattern in the spline curve for mortality [22]. Conversely, in patients with non-dialysis CKD and those undergoing intensive care with CKRT, a higher Cr/CysC ratio appears to correlate with a reduced risk of all-cause mortality [17,22,23].

In individuals with anuria undergoing dialysis, substantial alterations in serum creatinine levels aren’t anticipated due to glomerular filtration or tubular secretion. Nonetheless, numerous non-renal factors have been identified to impact creatinine and cystatin C concentrations. Foremost, when RRF diminishes, an increase in PD typically occurs to make up for insufficient dialysis, which may influence the kinetics of both serum creatinine and cystatin C. The main mechanism through which PD eliminates toxins involves diffusion, whereby the peritoneum acts as a semipermeable membrane facilitating solute transfer. As a small molecule with a high solute transport coefficient, serum creatinine is rapidly cleared through PD. Conversely, serum cystatin C, being larger and possessing a lower solute transport coefficient, is cleared less effectively by PD [24]. This study revealed that participants exhibiting a high Cr/CysC ratio showed reduced total Kt/V levels; furthermore, a negative correlation was found between the Cr/CysC ratio and total Kt/V, signaling that the connection between the Cr/CysC ratio and dialysis adequacy indicators remains a topic of debate. Additionally, PD is frequently associated with several cardiovascular risk factors. Recent research indicates that inflammation is a significant contributor to cardiovascular disease development in CKD patients [25,26]. Lower serum creatinine levels have been identified in HD patients who display hs-CRP and reduced serum albumin levels [27]. Furthermore, elevated levels of cystatin C have been found in diabetic individuals and those experiencing chronic inflammation [28]. Hence, the Cr/CysC ratio might also indicate the inflammatory or nutritional condition. This article identifies a significant correlation between the Cr/CysC ratio and BMI. However, due to the lack of data on patients’ muscle mass during the same period, the relationship between the Cr/CysC ratio and muscle mass remains largely speculative. Additionally, the correlations between the Cr/CysC ratio and hsCRP and albumin were not found to be significant. We hypothesize that this may be attributed to the cross-sectional nature of the collected variables, and we anticipate that these indicators will exhibit dynamic changes, including potential correlations, during follow-up. Additionally, the variability of the Cr/CysC ratio is crucial to consider, as it can be affected by the hydration status of the patient. This ratio is significantly influenced by factors such as body surface area, muscle mass, fat mass, and BMI. As a result, a reduction in RRF signifies not only impaired elimination of metabolic byproducts but also an elevated fluid burden.

The current research presents several merits. Firstly, to our knowledge, it is the inaugural investigation that highlights the association between the Cr/CysC ratio and both all-cause mortality and technique failure in anuric PD patients. Secondly, this retrospective analysis encompasses an extensive follow-up duration and meticulously explores the connection between the Cr/CysC ratio, dialysis adequacy, inflammation, and nutritional status. These variables are interconnected, and no earlier studies, as far as we are aware, have taken into account the presence of competing risks while examining the effect of the Cr/CysC ratio on these results. Thirdly, we utilized both qualitative and quantitative evaluations of the baseline Cr/CysC ratio, finding that its impact on all-cause mortality and technique failure in PD patients was consistent. Ultimately, the Cr/CysC ratio is easily accessible, economical, and possesses significant potential for clinical use. This investigation also has its limitations. Firstly, because it is retrospective, the independent association between the Cr/CysC ratio and all-cause mortality and technique failure should be interpreted with caution. Secondly, the sample size was relatively small, which hampers the broader applicability of the results; additional validation in a prospective, multicenter study is essential. Given the small sample size of this study, we did not further stratify the patients based on gender, age, or other factors. Thirdly, the findings may be affected by residual confounding, random errors, or uncontrolled variables, even after accounting for various potential confounders. Lastly, this research concentrated exclusively on the baseline Cr/CysC ratio, neglecting to analyze the effects of any changes in the Cr/CysC ratio over time on the outcomes, thus indicating a need for more extensive research in the future.

## Conclusion

In conclusion, the Cr/CysC ratio correlated with all-cause mortality and technical failure in anuric patients undergoing PD. Since the Cr/CysC ratio is an accessible and economical laboratory measurement, it may possess increased clinical relevance and potential for use in practice. Nonetheless, additional assessments are needed to evaluate its applicability across different populations.
